# Aortic Dissection in a Healthy Male Athlete: A Unique Case with Comprehensive Literature Review

**DOI:** 10.1155/2016/6460386

**Published:** 2016-09-21

**Authors:** Balraj Singh, Jennifer M. Treece, Ghulam Murtaza, Samit Bhatheja, Steven J. Lavine, Timir K. Paul

**Affiliations:** ^1^Department of Internal Medicine, Division of Cardiology, East Tennessee State University, Johnson City, TN, USA; ^2^Department of Internal Medicine, East Tennessee State University, Johnson City, TN, USA

## Abstract

A young otherwise healthy 27-year-old male who has been using anabolic steroids for a long time developed Type I aortic dissection associated with heavy weightlifting. The patient did not have a recent history of trauma to the chest, no history of hypertension, and no illicit drug use. He presented with severe chest pain radiating to back and syncopal event with exertion. Initial vitals were significant for blood pressure of 80/50 mmHg, pulse of 80 beats per minute, respirations of 24 per minute, and oxygen saturation of 92% on room air. Physical exam was significant for elevated jugular venous pressure, muffled heart sounds, and cold extremities with diminished pulses in upper and absent pulses in lower extremities. Bedside echocardiogram showed aortic root dilatation and cardiac tamponade. STAT computed tomography (CT) scan of chest revealed dissection of ascending aorta. Cardiothoracic surgery was consulted and patient underwent successful repair of ascending aorta. Hemodynamic stress of weightlifting can predispose to aortic dissection. Aortic dissection is a rare but often catastrophic condition if not diagnosed and managed acutely. Although rare, aortic dissection needs to be in the differential when a young weightlifter presents with chest pain as a delay in diagnosis may be fatal.

## 1. Introduction

Aortic dissection is a clinical emergency that commonly presents with tearing chest pain and hemodynamic instability. The immediate mortality rate in aortic dissection is as high as 1% per hour over the first several hours, making early diagnosis and treatment critical for survival. Therefore, high index of clinical suspicion is important as delay in diagnosis can have dreadful consequences. Aortic dissection in young healthy individuals has been reported in the literature but is relatively rare. In present review, we report a case of healthy male athlete with heavy weightlifting and use of anabolic steroids. He presented with chest pain in ER and was found to have aortic dissection.

## 2. Case Report

A 27-year-old Caucasian male was admitted to the hospital with the chief complaint of chest pain. On the day of admission, he went to the gym to lift weights, which he routinely does on a daily basis. During the workout, he developed severe chest pain with radiation to his back. He initially thought that he had pulled his muscle in his chest. After completing his weightlifting workout at the gym, he went to play basketball with his friends and continued to experience ongoing chest pain. On the basketball court, his friends noticed that he was holding his chest, was not feeling well, and reportedly had a syncopal event with exertion earlier in the day, so emergency medical services was called to bring him to the hospital. He described the chest pain as sharp, radiating to the back, and 10/10 in severity. The patient was cold, clammy, and short of breath. Prior to this incident, the patient had been healthy all his life without any medical problems. He never smoked, did not have hypertension, and had no family history of sudden cardiac death or any collagen vascular disease. There was no predisposing risk factor for aortic dissection other than heavy weightlifting. To develop and maintain a muscular body, he recently started injecting intramuscular anabolic steroids for the last 2 months and was going to the gym every day, lifting around 200 pounds at a time.

On examination upon arrival to the hospital, the patient's blood pressure was 80/50 mmHg, pulse was 80 beats per minute, respirations were 24 per minute, and oxygen saturation was 92% on room air. He was a well-developed, well-nourished, 5′9′′ muscular young male with a blue discoloration of his lips and extremities. Both upper and lower extremities were cold and clammy. The patient had diminished palpable radial pulses in his upper extremities but no palpable pulse in his lower extremities. His jugular venous pulse was elevated. Auscultation of his heart revealed distant, muffled heart sounds without any murmur. Lungs were clear to auscultation, and the patient was in mild respiratory distress with tachypnea. Abdominal examination was unremarkable. The patient was visibly uncomfortable, restless, and moaning in pain.

**Figure 1:**
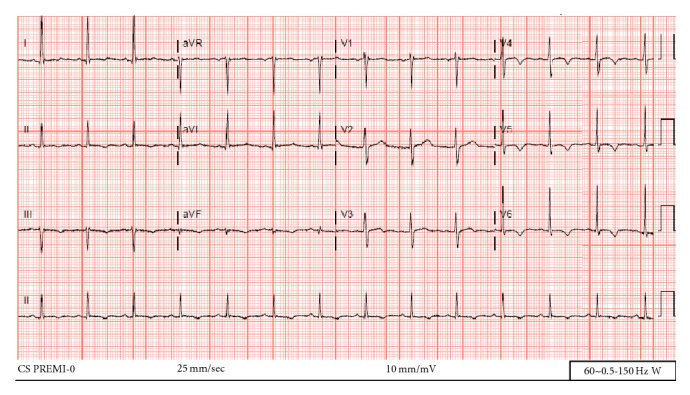
EKG suggests LVH and T-wave inversion in leads V4–V6.

**Figure 2:**
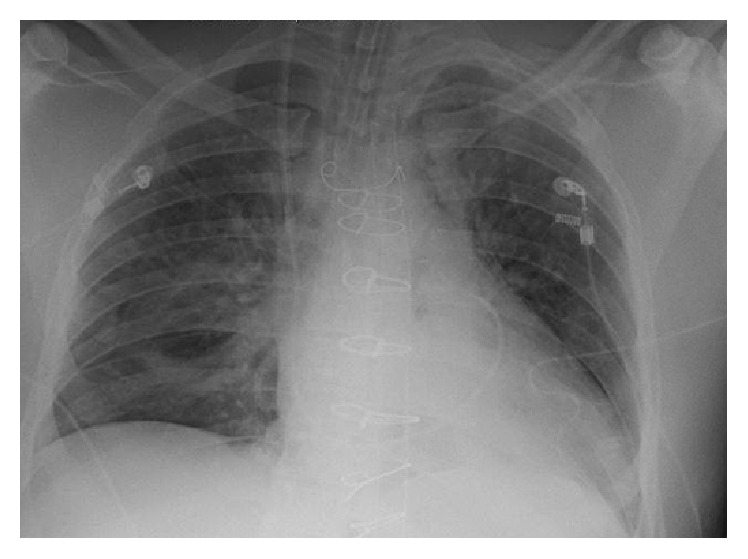
Chest X-ray: clear lungs, cardiomegaly, and wide mediastinum.

**Figure 3:**
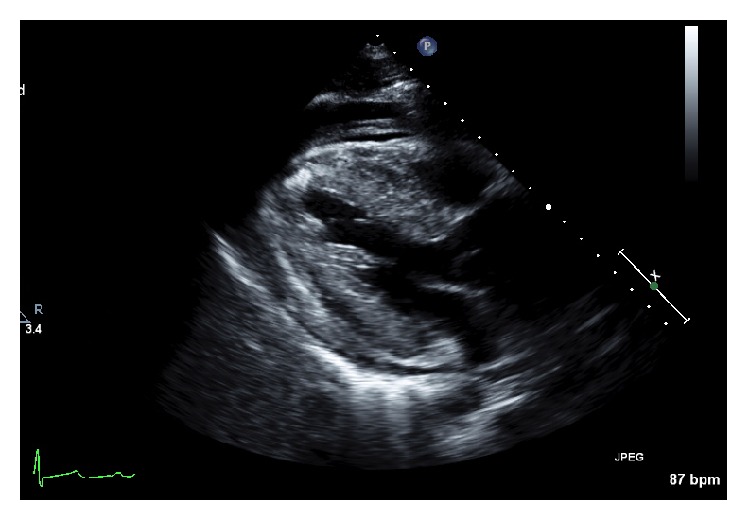
Echocardiogram showing left ventricular hypertrophy and pericardial effusion.

**Figure 4:**
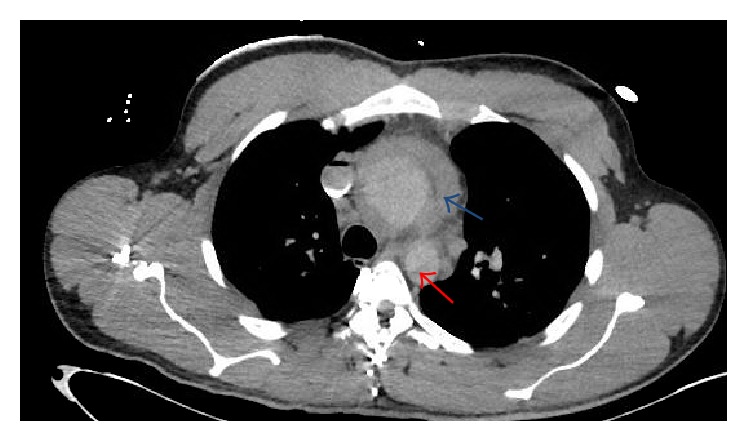
CT chest: ascending aortic dissection (red arrow) and hemopericardium (blue arrow).

**Figure 5:**
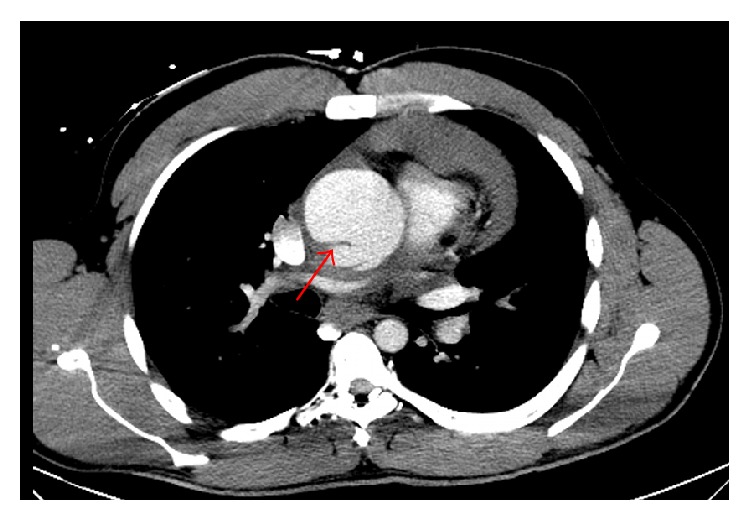
CT scan of chest with contrast showing dissection flap in ascending aorta (red arrow).

The patient's EKG revealed sinus tachycardia, nonspecific T-wave inversion in leads V4–V6, and voltage criteria suggesting LVH (Figure 1). The chest X-ray revealed clear lungs with cardiomegaly and a wide mediastinum (Figure 2). A STAT bedside echocardiogram was remarkable for moderate to severe left ventricular hypertrophy and a large circumferential pericardial effusion with cardiac tamponade and aortic root dilatation measured as 4.6 cm (Figure 3). A chest computed tomography (CT) immediately followed by echocardiogram revealed an ascending aorta measured as 5.7 cm in diameter with an aortic dissection suggesting rapid expansion of the dissection. Hemopericardium and cardiac tamponade were present with 2 cm thick fluid in the pericardium (Figures 4 and 5).

**Figure 6:**
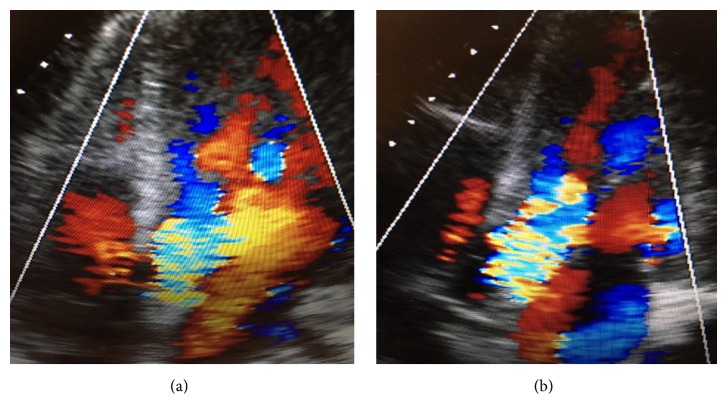
Echocardiogram five-chamber view showing severe aortic regurgitation.

A STAT cardiothoracic surgery consultation was done, and the patient was emergently taken to the operating room where a 28 mm Hemashield interposition aortic graft was placed. The aortic dissection had extended to the noncoronary cusp without involvement of the left main or right coronary arteries. It was challenging to repair the noncoronary cusp and keep the native valve intact. Intraoperatively, it was thought that the aortic regurgitation was acceptable given his age and to avoid lifelong anticoagulant for this young patient. Although the patient's initial echocardiogram prior to surgery showed no aortic regurgitation, the echocardiogram performed following the emergent repair of the ascending aortic dissection showed severe aortic regurgitation (Figures 6(a) and 6(b)). The patient's hospital course was complicated by persistent severe aortic regurgitation requiring a second procedure to repair the native aortic valve. Following the second surgical procedure, the patient's severe aortic regurgitation was minimized to moderate aortic regurgitation without necessitating aortic replacement. Both surgeries were successful for the patient. Pathology of the aortic specimen revealed cystic medial degenerative changes, which represent a loss of elastic fibers in the aortic media and atherosclerotic changes of aortic intima.

## 3. Epidemiology

Aortic dissection is a fatal condition with high morbidity and mortality. True incidence of this condition is usually underestimated due to the unknown contribution of aortic dissection to sudden deaths that occur out of the hospital setting as well as epidemiological studies of aortic dissection relying on collecting data retrospectively from hospital based registries. Reported incidence is 16 per 100,000 men and 7–9 per 100,000 women with the mean age at presentation of 63 years [1–3].

## 4. Etiology

There is a genetic predisposition for aortic dissection in cases of Marfan, Loey-Dietz, and Ehler-Danlos syndromes, familial aneurysms, bicuspid aortic valve, and coarctation of the aorta. Apart from genetics, the most common risk factors contributing to aortic dissection include hypertension, smoking, dyslipidemia, illicit drugs including cocaine and amphetamine, inflammatory disorders such as Takayasu, Behcet, and giant-cell arteritis, and trauma to the chest wall [4].

## 5. Pathology

The pathology of aortic dissection is not completely understood. Cystic medial degeneration is a noninflammatory loss of elastic fibers in the aortic media, and it is not pathognomonic for any of the abovementioned etiologies of dissection. Dissection usually starts with an intimal tear and is not always aneurysmal initially [5, 6]. Intramural hematoma and penetrating atherosclerotic ulcer can cause aortic dissection as well. Elevated blood pressure further propagates the intimal tear precipitating aortic dissection [5, 6].

## 6. Clinical Presentation and Diagnosis

Chest pain with radiation to the back is the most common complaint of a patient with aortic dissection, which was the initial presentation of the patient in this case report. Clinical scenarios for patients with aortic dissection may vary and can be challenging to diagnose depending upon the location of aortic dissection as the location dictates which other arteries and structures may also be affected. During an aortic dissection, there may be involvement of side branches of the aorta, which may cause malperfusion of the brain leading to a cerebral vascular accident, decrease blood to the heart muscle producing a myocardial infarction, or can involve adjacent structures and valves which may cause cardiac tamponade or aortic regurgitation. CT angiography scan is the preferred modality for diagnosis [7], and Transesophageal Echocardiogram may be needed if the patient is unstable or is not able to be transferred for a CT scan. Magnetic resonance angiography is another good alternative modality.

## 7. Treatment

Surgical treatment is recommended for Type A dissection. Overall in-hospital mortality is around 25%, and mortality for medically managed patients is 58%. Mortality is 1-2% per hour for the first day in patients who do not qualify for surgery [3, 7]. Surgery involves the placement of a synthetic interposition graft to reconstitute the true lumen. Surgery may or may not involve reimplantation of coronary arteries and resuspension of the native aortic valve, depending upon the level of dissection. Resuspension of the native aortic valve is preferred to replacement of the valve if possible [8].

## 8. Discussion

Aortic dissection in younger individuals is very rare. Excluding the patient discussed in this case study, as per our knowledge, there are only a few reported cases of aortic dissection in weightlifters with a history of anabolic steroid use, and one of these cases also had a history of cocaine and heroin use. The following list summarizes the six reported cases of aortic dissection in male weightlifters less than 38 years old [9–12].

*Brief Summary of Reported Cases of Male Weightlifters Less Than 38 Years Old Who Presented with Aortic Dissection*

*Type I Aortic Dissection*

1. 22 yo: no hypertension and unreported history of anabolic steroid use [11].
2. 37 yo: with hypertension and with a history of anabolic steroid use [11].
3. 37 yo: with hypertension, with a history of anabolic steroid use, and with history of cocaine and heroin use [11].
4. 34 yo: no hypertension and with a history of anabolic steroid use [12].

*Type II Aortic Dissection*

 28 yo: no hypertension, no anabolic steroid use, and no atherosclerosis [9].

*Type III Aortic Dissection*

 18 yo: no hypertension, no anabolic steroid use, and no atherosclerosis [10].

Pathology reports of all cases included cystic medial degeneration of the aortic tissue. Only the patient presented in this case report had pathology that also included atherosclerotic changes of aortic intima. This was the only patient with known current active anabolic steroid use at the time of aortic dissection.

Aortic dissection is categorized based on the location and origin of the dissection. Dissection of the ascending and descending aorta is defined as Type A and Type B dissection, respectively, as per Stanford classification. Aortic dissection is also classified as Types I to III as per DeBakey classification based on origination of the dissection. Type I aortic dissection has its origin in the ascending aorta and expands distally to the aortic arch and beyond the descending aorta. Type II aortic dissection originates in the ascending aorta where it remains confined without propagation beyond the ascending aorta. Type III aortic dissection has its origin in the descending aorta and expands distally [13]. All of the reported cases seen in the previous list are of aortic dissection in patients less than 38 years old who were active weightlifters with no genetic predisposition to development of aortic dissection, and pathology reports demonstrated cystic medial degeneration of the dissected aortic tissue in each case [9–12].

Our patient has been using anabolic steroids chronically that might be a contributing factor to aortic dissection but there is no clear association found between use of anabolic steroids and aortic root dilatation and dissection in the literature. More importantly he was likely having underlying mild aortic enlargement that concomitant with hemodynamic changes of heavy weightlifting raised aortic wall stress to a level that begets aortic dissection. Of the five reported cases of Type I aortic dissection in male weightlifters less than 38 years old, four of the patients confirmed use of anabolic steroids and the fifth patient had no known history of anabolic steroid use. Interestingly there are no reports of aortic dissection in anabolic steroids users that are not weightlifters. In the opposite there are quite a few reports of aortic dissection in weightlifters that do not use anabolic steroids. More importantly the pathological analysis does not suggest any scientifically proven relationship of steroids and atherosclerosis or of anabolic steroids and aortic dilatation or anabolic steroids and aortic dissection. Anabolic steroids theoretically could increase a patient's low density lipoprotein and decrease high density lipoprotein, thus promoting atherosclerotic deposition of the aortic intima and leading to weakening of the aortic wall. Therefore, atherosclerotic aortic walls are prone to dissection and rupture [9, 11]. The one Type II and one Type III aortic dissection cases did not have histories of anabolic steroid use. Although this case study shows that there is a correlation between anabolic steroid use and Type I aortic dissection in young male weightlifters, aortic dissection in this population is so rare that it is difficult to prove direct causation of anabolic steroid use leading to Type I aortic dissection. Weightlifting is known to elicit profound hemodynamic stress on the walls of the aorta [11], and weightlifting alone even without anabolic steroids use may predispose young patients to aortic dissection, particularly Type I aortic dissection. Although aortic dissection in this age group is very rare, it needs to be considered as a possible differential diagnosis in younger patients who are weightlifters with or without history of anabolic steroid use as the mortality and fatal morbidity are very high if left untreated [9].

The patient presented in this case had moderate to severe ventricular hypertrophy, which is difficult to explain in the absence of long standing hypertension. Anabolic steroid use may be the cause of his ventricular hypertrophy. A dilemma faced in the operating room for this patient was how to fix the aortic valve as the aortic dissection involved the noncoronary cusp. In this situation, the aortic valve could have been either replaced or repaired. Replacing the aortic valve would have committed this young patient to a lifetime anticoagulation therapy. On the other hand, repairing the noncoronary cusp would not require anticoagulants, but there would continue to be an acceptable degree of aortic regurgitation that has a potential to worsen over time, which was the initial option selected for this patient. Prior to aortic valve repair, the patient had severe aortic regurgitation, which was minimized to moderate aortic regurgitation following aortic valve repair, and the patient continues to remain asymptomatic to present day. He will likely need an aortic valve replacement in the future if he becomes symptomatic. The patient has been followed up as an outpatient with serial echocardiograms to reassess the degree of aortic regurgitation. He was strongly encouraged to avoid weightlifting and all anabolic steroid use. He continues to take lisinopril as an outpatient to decrease his afterload and therefore mitigate the effects of aortic regurgitation. He would have also been started on metoprolol had he not been bradycardic. He has been able to return to aerobic exercise without recurrence of symptoms and is seen regularly in the outpatient cardiology clinic.
